# Application of Response Surface Methodology Based on a Box-Behnken Design to Determine Optimal Parameters to Produce Brined Cabbage Used in Kimchi

**DOI:** 10.3390/foods10081935

**Published:** 2021-08-20

**Authors:** Hyeyeon Song, Eun-woo Moon, Ji-Hyoung Ha

**Affiliations:** Hygienic Safety and Analysis Center, World Institute of Kimchi, Gwangju 503-360, Korea; danbihy@wikim.re.kr (H.S.); luneeo@wikim.re.kr (E.-w.M.)

**Keywords:** brined cabbage, optimization, response surface methodology, salt gain, water loss

## Abstract

The factors of brine time, concentration, and temperature, affect the high-quality production of brined cabbage used in Kimchi. Although changes in Kimchi cabbage quality depending on brine time and concentration have been reported, changes in brine temperature have not been explored. Here, we optimized the brine process considering specific conditions of temperature (15–25 °C), concentration (10–14%), and osmosis duration (14–18 h) affecting the characteristics such as pH, titratable acidity, soluble solid content, glucose, fructose, and lactic acid bacteria and mass transport (salt gain, water loss, and weight reduction). The optimal parameters were determined using multivariate statistical analysis using the Box–Behnken design combined with response surface methodology. For each response as qualitative characteristics, second order polynomial models were developed using multiple regression analysis. Analysis of variance was performed to check the adequacy and accuracy of the fitted models. The brine temperature and concentration affected salt gain and water loss; the optimal brining temperature, concentration, and time were 19.17 °C, 10.53%, and 15.38 h, respectively. Statistical regression analysis indicated that standardized brined cabbage can be produced efficiently using a brining tank at controllable temperature.

## 1. Introduction

Kimchi is a traditional fermented food in Korea and is prepared by mixing brined Kimchi cabbage (*Brassica rapa* L. subsp. *pekinensis*) or various vegetables with seasoning mixtures such as radish, ginger, garlic, red pepper (*Capsicum annuum* L.) powder, and edible Allium varieties other than garlic [1]. In particular, Kimchi requires a lactic acid fermentation process at low temperatures to ensure microbiological safety, preservation, and proper ripening [1]. Many types of Kimchi are prepared depending on the vegetables used (e.g., cabbage Kimchi termed Baechu Kimchi, radish Kimchi termed Kkakdugi, cucumber Kimchi termed Oi-sobagi, and radish leaf Kimchi termed Yeolmu Kimchi), among which Baechu Kimchi is the most common and popular type in Korea and is also well known worldwide.

Brined cabbage is generally manufactured by the following process: Kimchi cabbage is trimmed and cut into halves, soaked in brine, washed, and drained to remove water [2,3]. During the brining process of Kimchi cabbage, osmotic pressure between salt and Kimchi cabbage inhibits the growth of microorganisms and creates an environment so lactic acid bacteria (LAB) that withstand high salt concentrations can dominate the fermentation in Kimchi [4]. Salt in food products can reduce water activity and cause high osmotic pressure on bacterial cells, which eventually inhibits microbial growth [5]. In addition, pectin is decomposed by enzymes associated with cell wall decomposition during the brining process. Furthermore, water-soluble substances such as vitamin C, sugar, sulfur-containing substances, and free amino acids are released from the fiber, which affect the taste [4] and texture [6]. Therefore, brining is considered an important process that influences quality characteristics such as taste, texture, and microbial growth of brined Kimchi cabbage.

Brined cabbage is an essential component of Kimchi and accounts for 70–90% of Kimchi ingredients [7,8,9]. Therefore, brined cabbage directly affects the quality properties of Kimchi, such as taste, flavor, and texture [10]. To improve the quality of brined cabbage, several studies have investigated the reducing efficacy of initial microorganisms using electrolyzed water [11,12] or ozonated water [13], quality improvement according to brining techniques [14,15] and type of salt [15]. In these studies, salinity, pH, titratable acidity, total sugar content, microorganisms, and texture were evaluated as indicators of the quality characteristics of brined cabbage. However, the influence of individual quality indicator factors on the quality of brined cabbage has not been explored. Moreover, the correlation between the quality index factors such as pH, titratable acidity, total sugar content, microorganisms, and texture, and the treatment condition factors regarding brining techniques remains unclear.

The brining process is affected by the brine time, brine concentration, and brine temperature, which are major factors influencing sensory quality [3,4,16]. Previous studies have investigated the change in quality characteristics of Kimchi cabbage according to brine time [16,17,18] and brine concentration [19,20], whereas there is a lack of studies regarding the effect of brine temperature. Moreover, there are no studies evaluating quality characteristics under the condition of a constant brine temperature during the entire salting process, although prior studies have examined the change in quality characteristics of Kimchi cabbage according to the initial brine temperature during the salting process [4,19]. According to Lee et al. [19], brine temperature fluctuates depending on numerous factors such as seasonal characteristics, brine tank size, and internal temperature of cabbage during the brining process. Therefore, in Kimchi manufacturing, the optimum conditions for brine temperature, brine time, and brine concentration are required as correlation factors for the quality of pickled cabbage.

The objective of this study was to identify the change in quality properties of prepared brined Kimchi cabbage according to brine time, brine concentration, and brine temperature, and to determine the optimal salting conditions. Response surface methodology (RSM) based on the Box–Behnken design (BBD) technique as a multivariate statistical analysis was used to define the optimum factors of the salting process and the correlation among experimental variables such as brine time, brine concentration, and brine temperature. Moreover, correlations between qualitative indicators (pH, titratable acidity, soluble solid contents, salinity, salt gain, water loss, weight reduction, and microbial growth) and treatment condition factors regarding brining techniques were investigated. Subsequently, we investigated the optimal salting conditions for manufacturing brined Kimchi cabbage.

## 2. Materials and Methods

### 2.1. Sample Preparation

Kimchi cabbage was obtained from an agricultural wholesale market (Gwangju, Korea). A solar salt (Sinan, Korea) was used to salt Kimchi cabbage. Kimchi cabbage was trimmed, cut in half, salted in brine, washed with water three times, and then drained for 3 h. At this time, the range for each independent variable was selected based on several previous reports [3,4,16,17,18]. The brined cabbage was prepared under various conditions depending on the brine time (14, 16, and 18 h), brine concentration (10%, 12%, and 14%), and brine temperature (15, 20, and 25 °C). Kimchi cabbage was placed in a self-made saline water tank (1650 mm (W) × 800 mm (L) × 750 mm (D), AsungTECH, Seoul, Korea), which was designed to maintain a constant temperature with a heater and chiller. Moreover, brine conditions were maintained so that all parameters were homogeneous during the pickling process using a saline water circulator. For the brine process, 200 L of brine solution, and 80 kg of fresh Kimchi cabbage were immersed. A custom-made temperature-controlled water bath was used in this study.

### 2.2. Experimental Design and Data Analysis

To determine the optimal factors, RSM was used for statistical analysis and correlation between experimental variables under the salting process. In this study, the key factors were the brine concentration (%), temperature (°C), and time (h) during the salting process. Minitab (Minitab software, LLC, State College, PA, USA) was used to design the experimental set using BBD, one of the most renowned methods. To obtain second-order polynomial regression models, we used a three-level BBD with three factors, which comprised the replicated central points and a set of points at the midpoint of each end of the multidimensional cube. The optimum experimental design was established using RSM based on BBD.

The three independent factors were investigated at three levels: 0 was the midpoint to determine the experimental error, while +1 and −1 were used for high and low levels, respectively (Table 1). To determine the optimal conditions of the three independent factors on the brining process of Kimchi cabbage, we used the second-order polynomial regression model (1):

(1) $$\left(Y\right)=\beta_{0}+\overset{k}{\underset{i=1}{\sum }}\beta_{i}X_{i}+\overset{k}{\underset{i=1}{\sum }}\beta_{ii}X_{i}^{2}+\overset{k}{\underset{i_{i=1}}{\sum }}\overset{k}{\underset{j>1}{\sum }}\beta_{ij}X_{i}X_{j}+\varepsilon$$

where *Y* is the response, X*_i_* and X*_j_* represent independent variables, and *β*_0_, *β_i_*, *β_ii_*, and *β_ij_* represent the constant, linear, quadratic, and interaction coefficients, respectively. *ε* signifies an error. A statistical analysis of variance (ANOVA) based on BBD was performed using Minitab to determine the fitness and suitability of the regression analysis coefficient. The significance of regression analysis was determined by various statistical factors, namely ANOVA, multiple determination coefficients (*R*_2_) tests, and a lack of fit test, provided by Minitab.

### 2.3. Measurement of Mass Transport

Water loss (WL), salt gain (SG), and weight reduction (WR) were investigated to explain the overall exchange of solute and water between Kimchi cabbage and brine. WL, SG, and WR were calculated using the mass balance Equations (2)–(4): WL is the net loss of water from Kimchi cabbage on an initial mass basis.

(2) $$\mathrm{WL}=\frac{W_{i}\cdot X_{i}-W_{\theta }\cdot X_{\theta }}{W_{i}}\times 100$$

SG is the net uptake of solids from Kimchi cabbage on initial mass basis

(3) $$\mathrm{SG}=\frac{W_{\theta }\left(1-X_{\theta }\right)-W_{i}\left(1-X_{i}\right)}{W_{i}}\times 100$$

WR is the net mass reduction of Kimchi cabbage on initial mass basis

(4) $$\mathrm{WR}=\frac{W_{i}-W_{\theta }}{W_{i}}\times 100$$

where *W_i_*: initial mass of Kimchi cabbage, g; *W_Ɵ_*: mass of brined cabbage after time *Ɵ*, g; *X_i_*: water content as a fraction of initial mass of Kimchi cabbage; *X_Ɵ_*: water content as a fraction of mass of brined cabbage after time *Ɵ*.

The water content of the homogenized brined cabbage was measured using an infrared moisture analyzer (Model MB 45; Ohaus, Pine Brook, Troy Hills, NJ, USA).

### 2.4. Measurement of Quality Characteristics

#### 2.4.1. pH, Titratable Acidity, and Soluble Solid Contents

The brined cabbage was homogenized and filtered to obtain juice. Titratable acidity and pH were measured using a digital pH meter (TitroLine 5000, SI Analytics, Mainz, Germany). For titratable acidity, 10 mL of filtrate was titrated until the pH reached 8.3, by adding 0.1 N NaOH solution, and calculated as the percentage of lactic acid. The soluble solid content (Brix) of the filtrate was measured using a refractometer (MASTER-20T, Atago, Japan).

#### 2.4.2. Salinity

Salinity was measured using the Mohr method [21], which determines the chloride ion concentration of a solution by titration with silver nitrate. K_2_CrO_4_ was an indicator during silver nitrate titration, and the appearance of red-brown precipitates was measured at the end of the assay. The samples were ground in a blender (HR1372, Koninklijke Philips N.V., The Netherlands), diluted with distilled water (1:20, *w*/*v*), and then filtered through filter paper (Hyundai Micro Co., Ltd., Seoul, Korea). In total, 10 mL of the filtered solution supplemented with 1 mL of 2% K_2_CrO_4_ solution as an indicator was titrated with 0.02 N AgNO_3_ solution until a red-brown color appeared. Salinity was calculated as salinity (%) = [A × F × 0.00117 × D/W] × 100, where A is the titration volume (mL) of 0.02 N silver nitrate; F is the factor of 0.02 N silver nitrate; D is the dilution factor; and W is sample weight (g).

### 2.5. Measurement of Free Sugars

The free sugar (glucose and fructose) content was determined using a modified Korea Food Code method. The samples were homogenized and filtered using sterilized acrylic gauze. Filtered samples were extracted with distilled water at 85 °C for 25 min. The extract was centrifuged at 3000× *g* for 10 min, filtered through a 0.2 μm syringe filter, and injected into the HPLC system (Model 1260 Infinity, Agilent Technologies, Santa Clara, CA, USA) with a refractive index detector. The mobile phase was 75% acetonitrile at a flow rate of 1.00 mL/min. The free sugars were separated on an Asahipak NH2P-50 4E column (4.6 × 250 mm, 5 μm, Shodex, Tokyo, Japan), which was maintained at 30 °C using a column oven. The injection sample volume was 10 μL. The concentrations are expressed in g/100 g of DW.

### 2.6. Comparison between Observations and Model Fittings/Predictions

The *Bf* value indicates how well the model predicts the measurement-mean variation for osmotic dehydration, whereas the *Af* value represents the scattering of the data. The deviation between the fittings/predictions and the experimental observations was estimated in terms of *Af* (Equation (5)) and *Bf* values (Equation (6)), which represent the bias factors and accuracy, respectively.

(5) $$A_{f}=10^{\left[\sum \frac{\left|\log\left(y_{predicted}/y_{observed}\right)\right|}{n}\right]}$$

(6) $$B_{f}=10^{\left[\sum \frac{\log\left(y_{predicted}/y_{observed}\right)}{n}\right]}$$

where *n* is the number of experimental cases, and *y* is WL or SG.

### 2.7. Measurement of Lactic Acid Bacteria

Samples (25 g) were homogenized with sterilized 0.85% saline (225 mL) in a Stomacher bag (Bagmixer 400, Interscience, Saint Nom, France) for 1 min, filtered through sterile cheese cloth, and diluted with 0.85% saline to measure microbial counts. LAB were determined using 3M Petrifilm LAB count plates (3M Microbiology, St. Paul, MN, USA). They were then incubated at 30 °C for 48 h. All experiments were performed in triplicate and the results were expressed as log CFU/g.

## 3. Results and Discussion

### 3.1. Statistical Analysis and Effect of Variables on Quality Characteristics

The values of responses in quality characteristics (pH, TA, SS, salinity, glucose, fructose, and LAB) and mass transport (SG, WL, and WR) according to brining conditions are presented in Table 2. The characteristics of the brined cabbage during the brining process varied depending on the operating conditions, such as time, temperature, and salt concentration. The pH and titratable acidity were 5.73–6.11 and 0.14–0.20%, respectively (Table 2). Similarly, the pH of conventional brined cabbage ranged from 5.27 to 6.47 (Kim et al., 2011) and the initial titratable acidity was below 0.2% [22]. The salinity of brined cabbage was between 1.81% and 3.55%, the level of typical brined cabbage. Jung et al. [23] demonstrated that the free sugar content of a seasoning mixture plays a major role in the flavor development of Kimchi by changing the composition of the cluster of LAB that give off sweet taste. However, for brined cabbage, no correlation between the free sugar content of brined cabbage and the LAB population according to the brining process was observed. For mass transport of brined cabbage samples, SG, WL, and WR were in the ranges of 0.45–3.29, 2.33–8.65, and 1.89–6.67, respectively.

**Figure 1:**
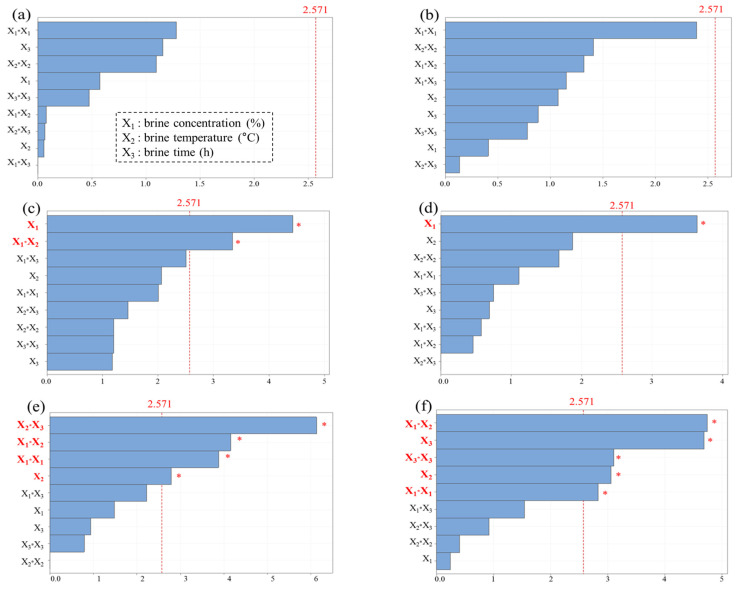

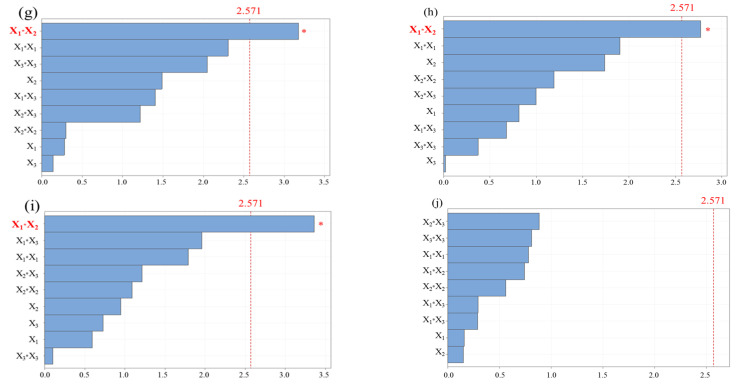
Pareto analysis of the efficacies of significant factors affecting the quality values on brined cabbage with statistical significance (*p* < 0.05). (**a**) pH, (**b**) titratable acidity, (**c**) soluble solid contents, (**d**) salinity, (**e**) salt gain, (**f**) water loss, (**g**) weight reduction, (**h**) fructose, (**i**) glucose, (**j**) lactic acid bacteria.

The Pareto analysis (Figure 1) shows the effect of brining variables (X_1_, X_2_, and X_3_) on the quality characteristics and mass transport of brined cabbage, which were generated and analyzed based on the data from the experimental results in Table 2. The Pareto chart showing the absolute value of the standardized effects is used to determine the magnitude and importance of the effects among the independent parameter effect, second-order effect, and interaction effect. Therefore, a Pareto chart illustrates the significance of each variable investigated in the experimental data and allows the primary effects of the factors to be ranked in order of their significance. The horizontal bar chart shows the calculated t-values, whereas the vertical line in the Pareto chart shows a table value of 2.571 for a 95% level of confidence based on first-order (X_1_, X_2_, and X_3_), second-order (X_1_*X_1_, X_2_*X_2_, and X_3_*X_3_), and interaction models (X_1_*X_2_, X_1_*X_3_, and X_2_*X_3_). SS was remarkably affected by the brine concentration and interactions of brine concentration (X_1_) and brine temperature (X_2_) (Figure 1c). In addition, interactions between brine concentration (X_1_) and brine temperature (X_2_) had a significant effect on the WR, fructose, and glucose of brined cabbage (Figure 1g–i). Although Lee et al. [19] reported that reducing sugar was not significantly affected by brine concentration, in this study, the interactions of brine concentration (X_1_) and brine temperature (X_2_) influenced fructose and glucose. The brine concentration had a significant effect on the salinity of the brined cabbage (Figure 1d). Our findings are similar to those of another study that reported the salinity of brined cabbage was primarily associated with brine concentration (Kim et al., 2009).

The changes in SG and WL as main osmosis parameters are dependent on the brining time, concentration, and temperature [17,18,19,20]. Therefore, the experimental data of SG and WL from brined cabbage samples were statistically analyzed by ANOVA based on the BBD to investigate the fitness and significance of the model coefficient among the first-order, second-order, and interaction models (Table 3). When analyzing the ANOVA results of two quality values, a high F-value with a low *p*-value (i.e., *p* < 0.05) indicates that the model is statistically significant, and these F- and *p*-values have a more significant influence on the corresponding model term toward the response variables [24]. Among the ANOVA results of SG reported in Table 3, the order in which the test variables contributed to the response was X_2_*X_3_ > X_1_*X_2_ > X_1_*X_1_. The Prob > F-value was observed to be less than 0.05, for X_2_*X_3_, X_1_*X_2_, and X_1_*X_1_, with F-values of 37.59, 17.28, and 15.03, indicating a significant model fit. The adequacy of the model was assessed using the coefficient of determination (R^2^) and lack of fit. The high R^2^ value and *p*-value for lack of fit higher than 0.05, indicate that the model was adequate. The R^2^ value was 94.48, indicating a good fit of the model to the data. In addition, the *p*-value for lack of fit was not significant (*p* = 0.106), suggesting that the fitted model predicted the experimental data well. Table 3 and Figure 1e show that SG was significantly affected by temperature in linear terms, brine concentration in quadratic terms, interaction between brine concentration and brine temperature, and interaction between brine temperature and brine time. Temperature is a well-known, significant parameter for osmosis, affecting the permeability of the cell membrane that allows solutes to penetrate by losing its selectivity [25]. For WL, the order in which the test variables contributed to the response was X_2_*X_3_ > X_1_*X_2_ > X_1_*X_1_. The Prob > F-value was <0.05, for X_1_*X_2_, X_3_, and X_3_*X_3_, with F-values of 22.52, 21.98, and 9.64, respectively. The R^2^ value and *p*-value for lack of fit were 93.93% and 0.303, respectively, showing a good fit between the experimental data and predicted data by the model. Table 3 and Figure 1f show that WL was significantly affected by brine temperature and brine time in linear terms, brine concentration and brine time in quadratic terms, and interaction between brine concentration and brine temperature. Ramya et al. (25) demonstrated that a higher brining temperature and immersion time lead to rapid water loss and solute uptake.

Generally, osmotic dehydration removes or reduces water from vegetables soaked in a hypertonic salt solution. During the osmotic process, water content trickles out from the fresh vegetables into the hypertonic salt solution, whereas the osmotic solute penetrates from the solution into the cellular tissue. Therefore, the brining process of cabbage is considered osmotic dehydration during Kimchi manufacture [4]. The level of water release from any vegetable made up of cellular tissue depends on variables such as the concentration and temperature of the salt solution, treatment time, material size, and material shape [26]. In this study, the order in which the independent variables contributed to the response of SG was brine temperature (°C) > brine concentration (%) > brine time (h) and of WL was brine time (h) > brine temperature (°C) > brine concentration (%). Previous studies have explored various salt concentrations, fixed process times, different process times based on specific salt concentrations, and different combinations of process time and salt concentration to define the optimal process conditions for producing high-quality Kimchi products [16,17,18,19]. Interestingly, our experimental data verified statistically that the condition of constant brine temperature is primarily related to the quality of the brined cabbage. This finding is invaluable to inform standardized production of brined cabbage, which would have a substantial effect on the quality of Kimchi obtained.

During osmotic dehydration, inappropriately high temperatures of hypertonic salt solutions can damage the quality of the vegetable due to thermal denaturation of proteins [27]. Furthermore, increased protein denaturation at a high brine temperature compared with a suitable brine temperature causes less osmotic dehydration, and low brine temperature inhibits osmotic dehydration [28]. Therefore, it is necessary to define the optimum constant temperature for all regions of the cabbage to be processed to treat an efficient time and brine concentration.

### 3.2. Response Surface Plots and Optimization of Brining Process Parameters in Kimchi Cabbage

The equation (Equation (1)) for a second-order polynomial regression model was fitted with the observed quality values for SG and WL, as presented in Table 2. The regression equation demonstrating the results of the response variables on the SG from the hypertonic salt solution in terms of the coded values of the variables (7) and (8):

*Y*_SG_ (salt gain) = 13.2 − 5.73 X_1_ − 0.727 X_2_ + 1.76 X_3_ + 0.1413 X_1_*X_1_ − 0.00004 X_2_*X_2_ − 0.0290 X_3_*X_3_ + 0.0582 X_1_*X_2_ + 0.0778 X_1_*X_3_ − 0.0859 X_2_*X_3_ (7)

**Figure 2:**
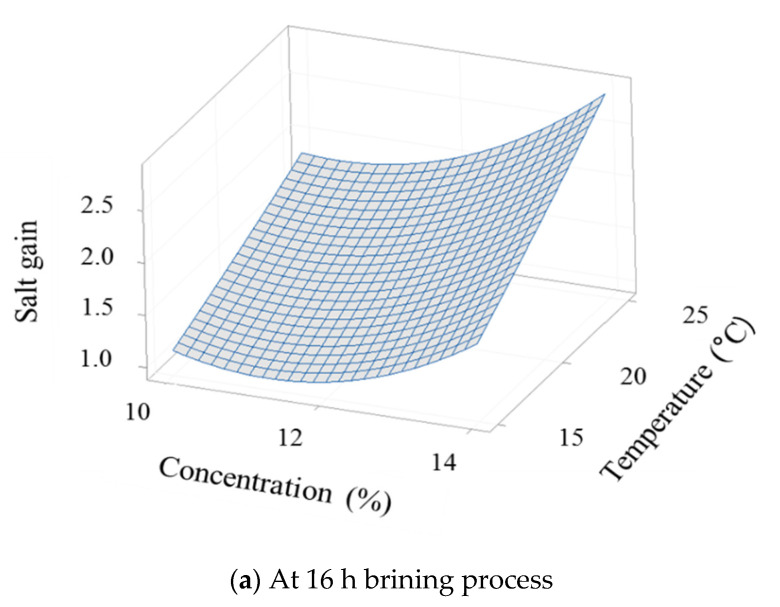

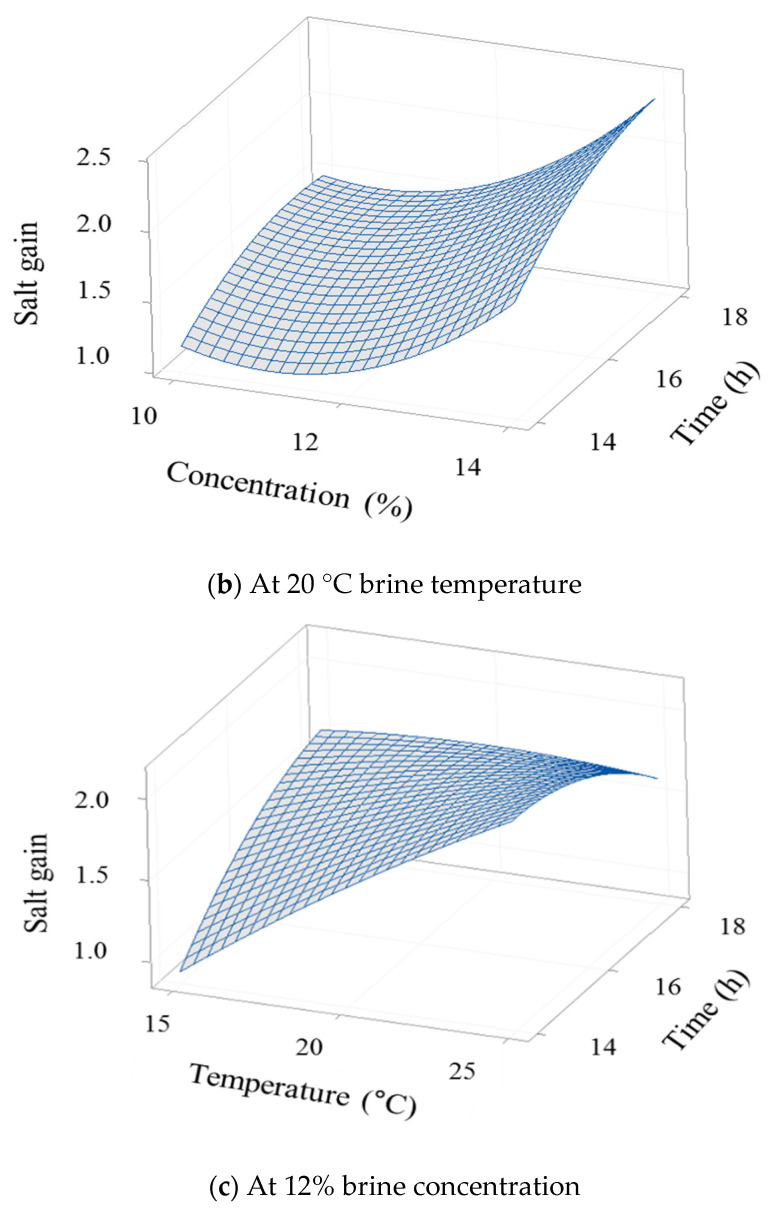
The 3D response surface plots for the salt gain during brining process of Kimchi cabbage as a function of (**a**) salt concentration and solution temperature, (**b**) salt concentration and immersion time, (**c**) solution temperature and immersion time.

**Figure 3:**
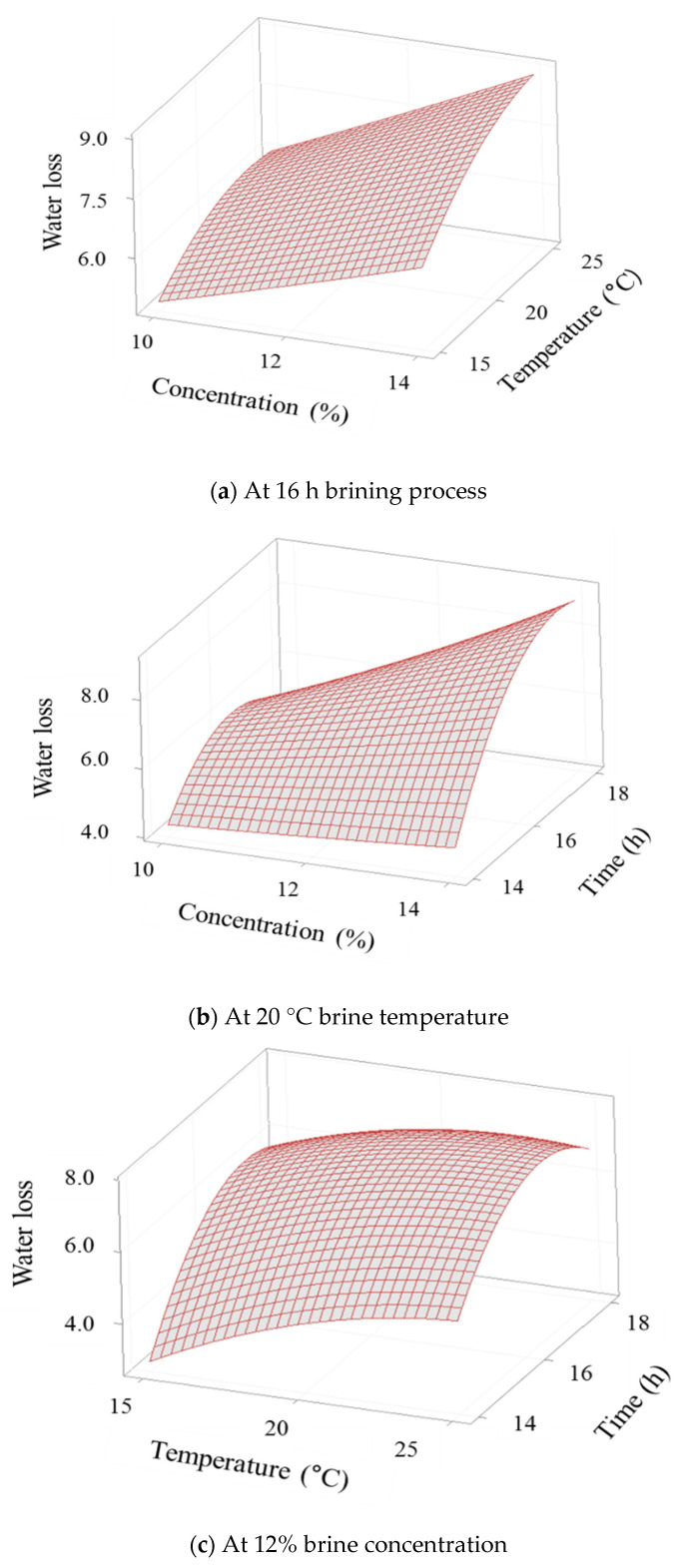
The 3D response surface plots for the water loss during brining process of Kimchi cabbage as a function of (**a**) salt concentration and solution temperature, (**b**) salt concentration and immersion time, (**c**) solution temperature and immersion time.

*Y*_WL_ (water loss) = 34.5 − 14.69 X_1_ − 0.209 X_2_ + 6.96 X_3_ + 0.4123 X_1_*X_1_ + 0.0057 X_2_*X_2_ − 0.2697 X_3_*X_3_ + 0.0157 X_1_*X_2_ + 0.2366 X_1_*X_3_ − 0.0309 X_2_*X_3_ (8)

where *Y*_SG_ and *Y*_WL_ refer to the SG and WL from hypertonic salt solution into the cellular tissue of cabbage, and X_1_, X_2_, and X_3_ are the uncoded values of the brine concentration (%), brine temperature (°C), and brine time (h), respectively. The 3D response surface plots were prepared for the fitted model to visualize the combined effect of the two variables on the SG (Figure 2) and WL (Figure 3). Temperature is associated with permeability of the cell membrane, and a decrease in solution viscosity by increasing temperature may affect SG by reducing resistance to diffusion of solute into the tissue [29]. The SG increased or decreased with brine time (Figure 2b,c). Delgado et al. [30] reported that increasing the osmotic dehydration time increases the mass transfer due to the cell membrane swelling and plasticizing effect, resulting in an increase in SG. The WL increased with the brine concentration (Figure 3a,b) and brine temperature (Figure 3a,c). In osmotic dehydration, brine concentration and temperature influence WL, and high brine temperatures promote WL by causing swelling and plasticization of cell membranes [29,30,31]. Delgado et al. [30] reported that the concentration was more significant than the temperature; however, in our study, the interaction between temperature and concentration was most significantly affected by WL.

Table 4 shows the optimal brining process parameters of the brining process according to the targeted SG and WL values. Recently, a trend of low-salt foods has appeared, and many studies have been conducted to develop low-salt Kimchi (Lee et al., 2002). In addition, the recommended sodium content of Kimchi was 790 mg/100 g in 2013, but it was reduced to 620 mg/100 g in 2016, and the goal was 480 mg/100 g in 2022 according to policy trends of sodium reduction [32]. The salinity of commercial Kimchi seasoning was between 1.73% and 5.35% [33], and it is difficult to control the salinity because Kimchi seasoning comprises various raw materials such as red pepper powder, garlic, onion, ginger, etc. Therefore, it is necessary to reduce the salinity of brined cabbage, and the target value of the SG is minimized. Therefore, the targeted values of SG and WL were determined to be the minimum (0.448) and maximum (8.65) values in our study, respectively. The optimal brining process conditions were brine concentration, temperature, and time at 10.53%, 19.17 °C, and 15.38 h, respectively.

To verify the validity of the obtained model based on the BBD, five experimental sets were performed under the optimized brining process conditions (Table 5). For the each optimized brining process, 200 L of brine solution, and 80 kg of fresh Kimchi cabbage were immersed and the optimum conditions were brine concentration, temperature, and time at 10.53%, 19.17 °C, and 15.38 h, respectively. Af and Bf take a value of 1, when the experimental observation values do not scatter and the fittings/predictions of the primary regression model perfectly match the means of the experimental data [33]. Bf values in the range of either >1.15 or <0.7 are considered unacceptable in the optimized model [34]. Bf values ranging from 0.7 to 0.9 or from 1.06 to 1.15 are considered acceptable in terms of the model, and those in the range from 0.9 to 1.05 are considered a good fit. For the five simulations under optimized brining process conditions, the two indices (*Af* and *Bf* values) derived from the observed and predicted values of WL and SG indicated goodness of fit and an acceptable model fit.

## 4. Conclusions

This study investigated the conditions for a typical brining process to produce standardized brined cabbage. The results indicated the optimal conditions were a brine temperature of 19.17 °C, brining concentration of 10.53%, and process time of 15.38 h. Statistical regression analysis suggested that standardized brined cabbage can be produced efficiently using a brining tank which can be controlled at a designated temperature, although the size of the brining tank was laboratory-scale. Optimal brining conditions are suggested, and it is expected that this information will be used for ensuring the quality of Kimchi through standardization of the cabbage brining process. However, further study is needed to determine if the optimized brine process is suitable for different cultivated varieties of Kimchi cabbage. Our approach makes a significant contribution to developing a practical brining process for the production of high-quality brined cabbage by determining optimal processing conditions.

## Figures and Tables

**Table 1:** Factors and levels used in the Box–Behnken design matrix for the brining process for the Kimchi cabbage.

Factors	Symbol	Levels		
		Low (−1)	Intermediate (0)	High (+1)
Brine concentration (%)	X_1_	10	12	14
Brine temperature (°C)	X_2_	15	20	25
Brine time (h)	X_3_	14	16	18

**Table 2:** Three-parameter BBD matrix with experimental quality values for the brining process for the Kimchi.

Run	Coded Values			Actual Values			Observed Quality Values									
	X_1_	X_2_	X_3_	Con ^(1)^	Tem ^(2)^	Time	pH	TA ^(3)^	SS ^(4)^	Salinity	Salt Gain	Water Loss	Weight Reduction	Fructose	Glucose	LAB ^(5)^
1	1	1	0	14	25	16	5.81	0.18	7.00	3.51	3.29	8.65	5.36	1.09	1.35	3.46
2	0	−1	−1	12	15	14	5.73	0.15	5.65	2.34	0.45	2.33	1.89	1.06	1.09	0.00
3	0	0	0	12	20	16	5.90	0.14	5.90	2.95	1.38	6.00	4.62	1.01	1.11	1.77
4	−1	0	−1	10	20	14	5.89	0.18	5.80	2.33	2.00	6.55	4.55	1.16	1.28	1.54
5	−1	0	1	10	20	18	5.95	0.19	5.70	1.81	1.78	6.62	4.84	1.23	1.20	0.00
6	0	0	0	12	20	16	5.94	0.16	5.73	2.78	1.50	6.05	4.55	1.07	1.20	1.47
7	0	1	−1	12	25	14	5.75	0.16	6.50	2.89	2.35	5.42	3.08	1.11	1.22	0.00
8	0	−1	1	12	15	18	5.89	0.14	5.90	3.00	2.14	5.41	3.28	1.04	1.20	3.33
9	1	0	−1	14	20	14	6.05	0.15	5.95	2.87	1.49	4.72	3.23	1.09	1.21	2.65
10	−1	1	0	10	25	16	6.07	0.16	5.45	2.62	1.65	7.45	5.80	0.92	1.00	3.39
11	1	0	1	14	20	18	6.11	0.20	7.05	2.75	2.52	8.58	6.06	1.31	1.61	0.00
12	−1	−1	0	10	15	16	6.11	0.17	6.05	2.09	2.00	8.06	6.06	1.43	1.56	2.72
13	0	0	0	12	20	16	5.98	0.17	5.55	2.60	1.62	6.10	4.48	1.12	1.30	0.00
14	1	−1	0	14	15	16	5.82	0.15	6.00	3.30	1.31	7.20	5.88	0.99	1.08	0.00
15	0	1	1	12	25	18	5.93	0.15	6.05	3.55	0.60	7.27	6.67	0.86	1.03	0.00

^(1)^ Concentration; ^(2)^ Temperature; ^(3)^ TA: titratable acidity; ^(4)^ SS: soluble solid content; ^(5)^ LAB: lactic acid bacteria.

**Table 3:** Matrix design results for the experiments performed according to the Box–Behnken experimental design for quality values of brined Kimchi cabbage.

Source		Salt Gain				Water Loss			
	DF ^(1)^	Adj SS ^(2)^	Adj MS ^(3)^	F-Value	*p*-Value	Adj SS	Adj MS	F-Value	*p*-Value
Regression	9	6.71023	0.74558	9.50	0.012	34.5245	3.8361	8.60	0.014
Linear	3	0.74015	0.24672	3.14	0.125	14.0087	4.6696	10.47	0.014
X_1_	1	0.17409	0.17409	2.22	0.197	0.0269	0.0269	0.06	0.816
X_2_	1	0.40656	0.40656	5.33	0.043	4.1797	4.1797	9.37	0.028
X_3_	1	0.06949	0.06949	0.89	0.390	9.8021	9.8021	21.98	0.005
Square	3	1.27796	0.42599	5.43	0.050	15.4819	5.1606	11.57	0.011
X_1_*X_1_	1	1.17951	1.17951	15.03	0.012	3.5833	3.5833	8.03	0.036
X_2_*X_2_	1	0.00000	0.00000	0.00	0.994	0.0751	0.0751	0.17	0.699
X_3_*X_3_	1	0.04959	0.04959	0.63	0.463	4.2974	4.2974	9.64	0.027
Interaction	3	4.69212	1.56404	19.94	0.003	5.0339	1.6780	3.76	0.094
X_1_*X_2_	1	1.35594	1.35594	17.28	0.009	10.0436	10.0436	22.52	0.005
X_1_*X_3_	1	0.38710	0.38710	4.93	0.077	1.0683	1.0683	2.40	0.182
X_2_*X_3_	1	2.94908	2.94908	37.59	0.002	0.3824	0.3824	0.86	0.397
Residual Error	5	0.39228	0.07846			2.2299	0.4460		
Lack of Fit	3	0.36414	0.12138	8.63	0.106	2.2250	0.7417	299.93	0.303
Pure Error	2	0.02814	0.01407			0.0049	0.0025		
*R* ^2^		94.48				93.93			

^(1)^ DF—degrees of freedom; ^(2)^ Adj SS—adjusted sum of square; ^(3)^ Adj MS—adjusted mean square.

**Table 4:** The optimum parameters for the brining process based on the standardized quality values of brined Kimchi cabbage.

Targeted Quality Values		Optimum Process Parameters		
SG	WL	Concentration (%)	Temperature (°C)	Time (h)
Minimum (0.448)	Maximum (8.65)	10.53	19.17	15.38

SG: salt gain, WL: water loss.

**Table 5:** Accuracy (*Af*) and bias factor (*Bf*) values for the second-order polynomial regression model fitted with the observed quality values for SG and WL under optimized brining process conditions.

Optimized Brining Process	Salt Gain		Water Loss	
	*A_f_*	*B_f_*	*A_f_*	*B_f_*
Simulation 1	1.033	0.977	1.124	0.918
Simulation 2	1.029	0.981	1.207	1.056
Simulation 3	1.088	0.928	1.526	1.334
Simulation 4	1.023	0.987	1.207	1.056
Simulation 5	1.081	0.924	1.526	1.334

## Data Availability

The data presented in this study are available in the article.
